# Hypoglycemic and hypolipidemic effects of *Lippia origanoides* Kunth in diabetic rats

**DOI:** 10.1002/fsn3.4162

**Published:** 2024-05-13

**Authors:** Vinicius Carvalho Miranda, Yago Luis Gonçalves Pereira, Allane Patricia Santos da Paz, Keyla Rodrigues de Souza, Márcia Cristina Freitas da Silva, Nilton Akio Muto, Patrick Romano Monteiro, Agenor Valadares Santos, Moises Hamoy, Maria das Graças Freire de Medeiros, Iolanda Souza do Carmo, Maria Eduarda Moraes Silva, José de Sousa Lima Neto, Vanessa Jóia de Mello

**Affiliations:** ^1^ Research, Teaching and Extension Laboratory in Clinical Analysis, Institute of Biological Sciences Federal University of Pará Belém Brazil; ^2^ Human and Environmental Toxicology, Tropical Medicine Nucleus Federal University of Pará Belém Brazil; ^3^ Center for the Valorization of Bioactive Compounds from the Amazon Federal University of Pará Belém Brazil; ^4^ Laboratory of Biotechnology of Enzymes and Biotransformation Federal University of Pará Belém Brazil; ^5^ Laboratory of Pharmacology and Toxicology of Natural Products, Institute of Biological Sciences Federal University of Pará Belém Brazil; ^6^ Laboratory of Technological Innovation and Entrepreneurship in Drug–LITE Federal University of Piauí Teresina Brazil; ^7^ Laboratory of Organic Geochemistry–LAGO Federal University of Piauí Teresina Brazil; ^8^ Department of Biology, Center for Biological and Health Sciences Federal University of Maranhão São Luís Brazil

**Keywords:** cardiovascular protector, diabetes, hypoglycemic, *Lippia origanoides* Kunth

## Abstract

Diabetes mellitus is a metabolic disorder commonly associated with atherosclerosis. Plants with therapeutic potential, such as *Lippia origanoides* Kunth, emerge as effective alternatives for treating these diseases. Therefore, this work aims to analyze the antihyperglycemic and antidyslipidemic potential of the hydroalcoholic extract of *Lippia origanoides* Kunth (ELo) in alloxan‐diabetic rats. Animals were treated orally: normal control, hyperglycemic control, positive control glibenclamide (5 mg/kg), and groups treated with ELo (75, 150, and 250 mg/kg). Preclinical evaluation of ELo showed hypoglycemic, hypolipidemic, hepatic, and renal protective effects. At all doses, ELo significantly reduced hyperglycemia, triglycerides, total cholesterol, low‐density lipoprotein, atherogenic index, atherogenic coefficient, and cardiovascular risk index (*p* < .05). Elo at different doses promoted an increase in insulin release compared to untreated animals (*p* < .05) and showed α‐glucosidase inhibitory activity (*p* < .05). Also, ELo (250 mg/kg group) showed maximum reduction of hyperglycemia, alanine transaminase, aspartate aminotransferase, malonaldehyde, and urea compared to the hyperglycemic and glibenclamide groups, and creatinine only compared to the hyperglycemic groups (*p* < .05). The promising action of ELo in the context of diabetes may be related to the synergistic action of flavonoid compounds identified in liquid chromatography, whose pharmacological capabilities have already been documented in previous studies. The mechanisms may be the stimulation of insulin release; the inhibitory activity of α‐glucosidase; improving general clinical conditions; and the antioxidant effects of the extract. These findings pave the way for the future development of an herbal presentation of *L. origanoides* Kunth as a hypoglycemic and cardiovascular protector with a lipid‐lowering effect.

## Graphical abstract

This work evaluates the hypoglycemic and hypolipidemic effects of the hydroalcoholic extract of *Lippia origanoides* Kunth (ELo) in rats with alloxan‐induced diabetes. ELo demonstrated promising action in diabetes with a possible cardiovascular protective lipid‐lowering effect, serving as a basis for the development of toxicological studies that validate the use of this plant in traditional medicine and its introduction in future investigations in clinical studies with humans.

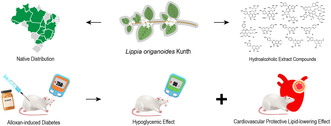

## 1 INTRODUCTION

*Lippia origanoides* Kunth (Verbenaceae) is a promising oregano‐like herb, known in northern Brazil as “*alecrim‐pimenta*,” “*salva‐do‐marajó*,” or “*alecrim‐d'angola*” (Castilho et al., 2019; Sarrazin et al., 2015). Aerial parts of this bushy species are used in regional cuisine as flavoring agents (Sarrazin et al., 2015). Within the genus *Lippia (L*.), it is described that *Lippia nadiflora* (Balamurugan & Ignacimuthu, 2011) and *Lippia javanica* (Arika et al., 2015) reveal promising anti‐hyperglycemic action, but this effect in *L. origanoides* Kunth has not yet been described experimentally.

Traditional and herbal medicines are receiving considerable attention from leading international health, research, and medical education authorities, given that around 80% of the world's population used traditional medicine in March 2022, with 170 of the 194 WHO Member States declaring support for its use due to its effectiveness, safety, and accessibility (WHO, 2022). In addition to the benefits of herbal medicine for humans (Hao et al., 2017; Khan et al., 2021; Mubashir et al., 2022), medicinal plants as food additives have economic benefits in animal health (Hegazy et al., 2023) and agriculture (Shahbaz et al., 2022). Among phytotherapy applications, more and more natural components are used as antidiabetics (Xu et al., 2018).

Diabetes is a metabolic disorder characterized by a chronic hyperglycemic condition resulting from defects in secretion and/or insulin action (Poznyak et al., 2020). The establishment of hyperglycemia is due to (1) insulin resistance due to decreased glucose uptake in skeletal muscle, increased glucose production in the liver, and increased inflammation and lipolysis in adipose tissue; and (2) insufficiency of pancreatic islets due to decreased function and mass of β‐cells (insulin‐secreting) and an increase in the function of α‐cells (glucagon‐secreting) (Javeed & Matveyenko, 2018).

Diabetes prevalence is rapidly increasing worldwide. According to the International Diabetes Federation, the adult population (20–79 years old) living with this disease was more than half a billion people in 2021 (537 million) (IDF Diabetes Atlas, 2021), with projections indicating that this number will more than double by 2050, reaching 1.31 billion people worldwide (GBD 2021 Diabetes Collaborators, 2023). Diabetes mellitus and atherosclerosis are linked through several pathological pathways, which has drawn attention to the coexistence of dyslipidemia and diabetes in recent years since diabetic patients are at increased risk of the onset and accelerated development of atherosclerosis (Hirano, 2018).

Although several conventional pharmaceutical therapies can help regulate blood glucose levels and manage diabetes symptoms, allopathic treatment has undesirable side effects, including gastrointestinal reactions (nausea, vomiting, diarrhea, and abdominal discomfort) and, more seriously, lactic acidosis – in diabetics with liver and kidney disease, circulatory problems, congestive heart failure, and in the elderly (DeFronzo et al., 2016; Dyatlova et al., 2022; Mohri et al., 2023). Therefore, this work aimed to evaluate the potential hypoglycemic and lipid‐lowering cardioprotective effects of the hydroalcoholic extract of *L. origanoides* Kunth (ELo) in hyperglycemic rats.

## 2 MATERIALS AND METHODS

### 2.1 Plant material

The aerial parts of the plant (*L. origanoides* Kunth) were collected during flowering (May) from José de Freitas District (latitude 04°45′23″ south and longitude 42°34′32″ west), Piaui, Brazil, 2019. The voucher specimen was deposited and taxonomically identified in the herbarium of “Graziela Barroso” of the Federal University of Piaui Biology Department. TEPB 9.205. Registered in Sisgen‐MMA Brazil‐Sisgen N°‐ AB30D19.

### 2.2 Hydroalcoholic extract of *Lippia origanoides* Kunth: preparation and chemical characterization

The standardized extract was obtained according to Coelho et al. (2015). As proposed by the authors, 900 grams of fresh plant material were dried in a forced‐air oven at a temperature between 50°C and 60°C for 6 hours until a constant dry mass was obtained.

The hydroalcoholic extract was obtained from 270 grams of dried aerial parts of *L. origanoides* Kunth subjected to maceration in ethanol/water (1:1) for 3 days; the hydroalcoholic extract was filtered and concentrated in a Fisatom® 802 rotary evaporator (coupled to a vacuum pump 826 T®‐Fisatom®, 50°C, cooling at 11°C under a pressure of 600 mmHg), and subsequently dried in a lyophilizer L101 (Liotop®); the dry extract was later homogenized in a mortar and kept in a desiccator until constant mass.

The characterization of ELo was performed by LC–MS, Amazon Speed ETD–Bruker® coupled to a Shimadzu® liquid chromatography system with 30 AD pumps at a flow rate of 1 mL/min, an SPD‐20A detector with a selected wavelength of 288 nm, CTO‐Over 20A for column (Phenomenex Luna C18 250 × 4.6 mm; 5 μm) at room temperature, and SIL‐30 AC autosampler with an injection volume of 10 μL. Chromatographic analysis was performed using Water (Solvent A) and Methanol (Solvent B) solvents in a gradient method starting at 5% B, maintained for 2 min, and reaching 100% in 62 min of analysis and 100% B maintained for up to 77 min.

ELo was analyzed by mass spectrometer performed analysis by electrospray ionization, and the multistage fragmentations were obtained in an ion trap analyzer in negative mode with a capillary voltage of 4.5 kV, a capillary temperature of 300°C, N_2_ carrier gas (12 L. min^−1^), a nebulizer gas pressure of 30 psi, and an acquisition range of *m/z* 100–1000. The identification of the constituents was based on the multistage fragmentation profile, comparison to a database of MS/MS spectra like the Massbank® (https://massbank.eu/MassBank/), and a literature review.

### 2.3 Experimental animals

The study used male Wistar albino rats (6–8 weeks old) weighing 200–250 g. These were raised in the vivarium of the Institute of Biological Sciences of the Federal University of Pará‐UFPA and housed in air‐conditioned rooms in plastic cages with free access to drinking water and chow (standard commercial diet Labina; Purina®, São Paulo, Brazil: 56.81% carbohydrates, 23.27% protein, 4.24% fat, and 3584 kcal/g), under controlled conditions of humidity (50 ± 10%), light (12/12 h light/dark cycle), and temperature (25 ± 2°C).

#### 2.3.1 Ethical statements

This study began after approval by the local Committee for the Use and Care of Experimental Animals of the Federal University of Pará, Belém‐Brazil (CEUA n° 82,113,001,200). In addition, the guidelines postulated in the National Research Council Guide‐US (Guide for the Care and Use of Laboratory Animals) were adopted, and the experiments were carried out following the National Council for the Control of Animal Experimentation (CONCEA).

### 2.4 Hydroalcoholic extract of *Lippia origanoides* Kunth dose

The dose of Elo was optimized based on the evaluation of similar studies with plants of the same genus, *L. nadiflora* (Balamurugan & Ignacimuthu, 2011) and *L. javanica* (Arika et al., 2015), which demonstrated hypoglycemic activity in the range of 50–200 mg/kg of body weight. In a pilot study with normoglycemic animals, considering the oral administration of ELo, a dose of 150 mg/kg was chosen for the oral glucose tolerance test, opening the dosage range to 75 mg/kg, 150 mg/kg, and 250 mg/kg of ELo in subsequent tests.

### 2.5 Induction of hyperglycemia

After 7 days of acclimatization, chronic hyperglycemia was induced in the rats, after fasting overnight (12 h), with a single intraperitoneal (i.p.) injection of freshly prepared alloxan monohydrate (2,4,5,6 tetraoxypyrimidine;5‐6‐dioxyuracil) obtained from Sigma® (Steinheim, Switzerland), dissolved in ice‐cold saline at a dose of 120 mg/kg (body weight). Rats with fasting glucose above 250 mg/dL after 72 h of hyperglycemic induction were chosen for the study, according to Lee and McCarty (1990) and Ahiskali et al. (2019). Blood was collected from the caudal end, and fasting blood glucose was analyzed using a glucometer‐Accu check® (Elangovan et al., 2019). After confirmation of the hyperglycemic state, the animals were distributed into three experimental blocks (*n* = 84): the first composed of groups in which the oral glucose tolerance test was performed; the second block consisted of animals treated with repeated doses of the extract for up to 15 days (Balamurugan & Ignacimuthu, 2011); and the third block consisted of animals with treatment extended to 28 days (Elangovan et al., 2019). The three blocks analyzed the hypoglycemic potentials of ELo, and these experimental steps are better described in the following topics.

### 2.6 Experimental design for oral glucose tolerance test

The oral glucose tolerance test examined the animals' ability to tolerate the glucose load. For this, after an overnight fast (12 h), 3 groups of hyperglycemic animals received (orally) the following pre‐treatments (*n* = 8/group):

- Group 1: Received only vehicle (water).
- Group 2: Received 150 mg/kg of ELo.
- Group 3: Received 5 mg/kg of glibenclamide.

30 min after pre‐treatments, the animals received (orally) a single dose of glucose solution (2.0 g/kg of body weight). Blood glucose levels were measured at 0 (just before glucose ingestion), 30, 60, 90, and 120 min after glucose solution ingestion (blood collection and glucose measurement similar to those described in Section 2.5).

### 2.7 Experimental design for 15 and 28 days of treatment

Initially, experimental animals were categorized into six groups (*n* = 10/group):

- Group 1: The normoglycemic group that received only vehicle (water).
- Group 2: Hyperglycemic control with alloxan‐induced hyperglycemia, which received vehicle.
- Groups 3, 4, and 5: Alloxane‐induced hyperglycemic rats that received daily ELo 75, 150, and 250 mg/kg, respectively.
- Group 6: Alloxane‐induced hyperglycemic rats that received a daily oral dose of 5 mg/kg of glibenclamide.

After 15 days of treatment and overnight fasting (12 h), half of the animals in each group (*n* = 5/group) were euthanized (by ketamine/xylazine 300/30 mg/kg i.p.) for immediate blood collection for biochemical analysis (lipid, renal, hepatic profile, and malonaldehyde (MDA) dosage) and collection of liver and pancreas for histological analysis. Previously, body weight and fasting blood glucose had been estimated at 0, 7, and 15 days of treatment (blood collection and fasting blood glucose measurement were similar to those described in Section 2.5). All results were compared with values obtained by animals from the normoglycemic group, hyperglycemic control rats, and the glibenclamide group.

The other half of the animals (*n* = 5/group) continued receiving treatment until reaching 28 days. Fasting blood glucose was estimated at 21 and 28 days. At the end of treatment, the animals were euthanized (by ketamine/xylazine 300/30 mg/kg i.p.) after overnight fasting (12 h). Blood was immediately collected to measure insulin and glycated hemoglobin (HbA1c). All results were compared with the values obtained from animals in the normoglycemic, hyperglycemic control, and glibenclamide groups.

#### 2.7.1 Serum biochemical parameters

##### Lipid profile, kidney function, and liver function

The serum was separated by centrifuging blood at 3000 rpm for 10 min and was subjected to biochemical analysis for estimation of serum lipid profile (total cholesterol, triacylglycerides, low‐density lipoprotein (LDL), and high‐density lipoprotein (HDL)–mg/dl). The atherogenic index, atherogenic coefficient, and cardiovascular risk index were calculated using the formula by Harnafi et al. (2008), Kinosian et al. (1994), and Azizian et al. (2018), respectively:

$$\text{Atherogenic Index}=\frac{\;\left(\text{Total Cholesterol}–\mathrm{HDL}\;\text{Cholesterol}\right)}{\mathrm{HDL}\;\text{Cholesterol}}$$

$$\text{Atherogenic Coefficient}=\frac{\mathrm{LDL}\;\text{Cholesterol}\;}{\mathrm{HDL}\;\text{Cholesterol}}$$

$$\text{Cardiovascular Risk Index}=\frac{\text{Total Cholesterol}\;}{\mathrm{HDL}\;\text{Cholesterol}}$$

Serum parameters of kidney function, such as urea and creatinine values, and liver function, such as alanine transaminase (ALT) and aspartate aminotransferase (AST), were evaluated. These parameters were estimated (mg/dl) using commercial kits from Wiener Lab® with the Automatic Clinical Chemistry Analyzer CM200 Wiener®.

##### Oxidative stress assessment

The thiobarbituric acid‐reactive substances (TBARS) were determined as described by Ohkawa et al. (1979) and Pyles et al. (1993). This test indicates the presence of products derived from lipid peroxidation, mainly MDA. Plasm 60 μL was recovered with 15% trichloroacetic acid, and the supernatant was incubated with 10 mM thiobarbituric acid and heated at 92°C for 120 min. The resulting lightly pink‐colored product was placed in a 96‐well microplate for spectrophotometer quantification at 535 nm (BIO‐RAD Model 450 Microplate Reader). The results are expressed in μmol MDA.

##### Measurement of insulin and HbA1c

The serum samples at 28 days of ELo treatment were used to estimate insulin levels by Direct Chemiluminescence method–Competitive Immunoassay, as described by Kumar et al. (2016). The dosage of HbA1c was obtained by HPLC (High‐Performance Liquid Chromatography).

#### 2.7.2 Histopathological studies

The pancreas and liver from experimental animals were fixated with 10% formalin and paraffin for embedding the tissues. Thick tissue sections (5 μm) were processed and subjected to hematoxylin and eosin (H&E) staining.

### 2.8 Inhibitory activity against α‐Glucosidase

The methodology used was based on and adapted from the methods described by Schmidt et al. (2012). The enzyme α‐glucosidase of *Saccharomyces cerevisiae* was resuspended in phosphate buffer 50 mM pH 7.0 at a final concentration of 1 mg/mL and then stored at −20°C until use. The para‐nitrophenyl‐alpha‐glycoside substrate (pNPG‐α) was resuspended in the same buffer at a final concentration of 20 mM and stored under the same conditions. As a stop reaction, the Na_2_CO_3_ solution at 1 M is used. For the assay, the enzyme was first diluted to generate activity of 0.04 U/mL in the reaction solution from the phosphate buffer (10 mM, pH 7.0). The reactional assay of a final volume of 100 μL was used, consisting of 25 μL of the enzyme, 30 μL of phosphate buffer 100 mM pH 7.5, and 20 μL of extract, which will be incubated for 15 min at 30°C. Subsequently, 25 μL of pNPG‐substrate was added to the reaction and incubated for 30 min at 30°C. The stop agent (100 μL) has been added, and then the solution will be read at 405 nm in the spectrophotometer. A solution containing 50 μL of buffer, 25 μL of the enzyme, and 25 μL of the substrate was used for the control. The enzymatic activity of α‐glucosidases was calculated from the formula:

$$\text{Enzymatic Activity}\;\left(U/\mathrm{ml}\right)=\frac{\left[\left(\left|\mathrm{Abs}\;\text{test}\right|\right)-\left(\left|\mathrm{Abs}\;\text{blank}\right|\right)\right]*\mathrm{Vt}*\mathrm{fd}}{18,1*t*\mathrm{Vs}.}$$

Abs: Absorbance measured at 405 nm.

Vt: Total volume (200 μL).

fd: Dilution factor.

18.1: Molar para‐nitrophenol extinction coefficient in cm^2^/micromolar.

t: Reaction time (30 min).

Vs: Reaction volume (100 μL).

### 2.9 Statistical analysis

The statistical analyses were performed using the statistical package *GraphPad Prism* version 9. Kolmogorov–Smirnov test (Normality Test, *α* = .05) and analyses of variance were performed by ANOVA procedures, and the significance of each group was verified with Tukey's or Dunnett's post hoc *test* (*p* < .05), with values expressed as mean **±** SD.

## 3 RESULTS

### 3.1 Chemical profile of ELo by HPLC‐PDA‐ESI‐IT‐MS/MS^n^

**FIGURE 1:**
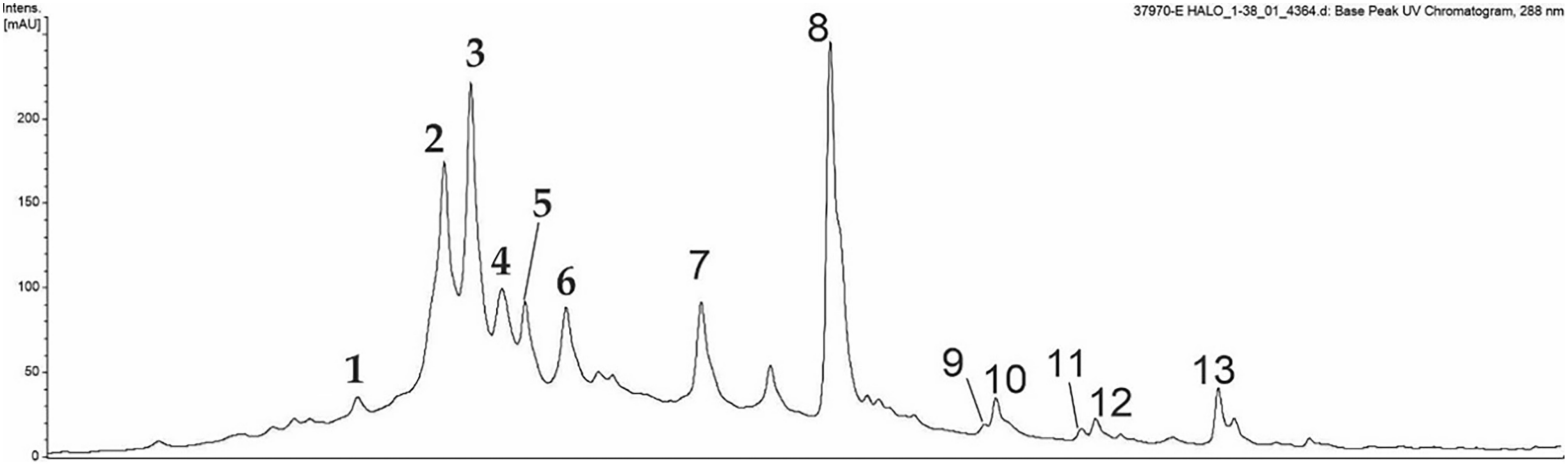
Chromatogram of hydroalcoholic extract of *Lippia origanoides* Kunth (ELo) obtained by HPLC‐PDA‐ESI‐IT‐MS/MS^n^ with UV detector at 288 nm. Peak numbers are identified in Table 1 and Figure 2. Experimental conditions are described in the Materials and Methods.

**FIGURE 2:**
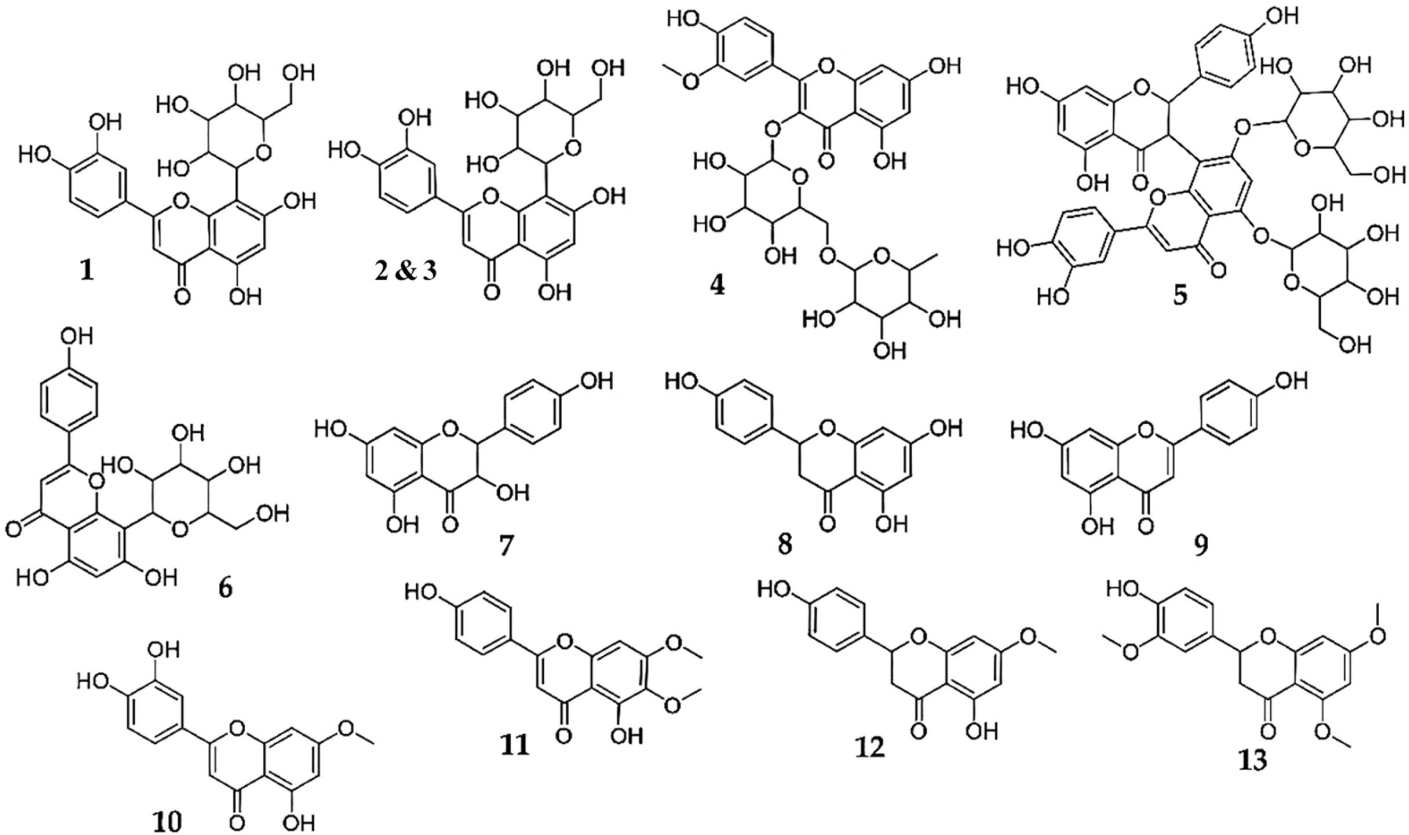
Chemical structures identified in the chromatogram of the hydroalcoholic extract of *Lippia origanoides* Kunth (ELo).

The HPLC‐PDA‐ESI‐IT‐MS/MS^n^ analyses of ELo showed a flavonoid profile (Figure 1). Using absorbance at 288 nm and total ion chromatogram, typical of flavonoid (Table 1, Figure 2), flavones such as apigenin, orientin, and metoxiflavones were identified. Moreover, ELo showed the presence of flavanones such as Naringenin, Cirsimaritin, and Hidroxytrimetoxiflavanone.

**TABLE 1:** Compounds identified in the hydroalcoholic extract of *Lippia origanoides* Kunth (ELo) by HPLC‐PDA‐ESI‐IT‐MS/MS^n^.

Peak	R_T_ (minutes)	[M‐H]‐	Fragment ions [MS^n^] (relative abundance, %)	Estimated identification
1	21.90	593	[593, **MS** ^**2**^]: 575, 503, 473 (100), 383, 353; [593 → 473, **MS** ^**3**^]: 583, 353 (100); [593 → 473 → 353, **MS** ^**4**^]: 325 (100), 297, 191; [593 → 473 → 353 → 325 **MS** ^**5**^]: 297 (100)	Vicenin‐2 (apigenin‐6,8‐di‐C‐glucoside)
2	24.30	447	[447, **MS** ^**2**^]: 429, 357, 327 (100); [447 → 327, **MS** ^**3**^]: 299 (100), 283, 191; [447 → 327 → 299, **MS** ^**4**^]: 213, 147	Orientin
3	25.00	447	[447, **MS** ^**2**^]: 429, 357, 327 (100); [447 → 327, **MS** ^**3**^]: 299 (100); [447 → 327 → 299, **MS** ^**4**^]: 271, 225, 188	Orientin (isomer)
4	25.80	623	[623, **MS** ^**2**^]: 461 (100); [623 → 461, **MS** ^**3**^]: 391, 315 (100), 135; [623 → 461 → 315, **MS** ^**4**^]: 179, 135 (100); [623 → 473 → 315 → 135, **MS** ^**5**^]	Isorhamnetin‐3‐O‐rutinoside
5	26.40	879	[915, **MS** ^**2**^]: 879 (100); [915 → 879, **MS** ^**3**^]: 717 (100), 591, 269; [915 → 879 → 717, **MS** ^**4**^]: 565, 447, 357, 327, 269 (100)	Morelloflavone‐4, 7‐di‐O‐hexose
6	27.50	431	[431, **MS** ^**2**^]: 413, 341, 311 (100); [431 → 311, **MS** ^**3**^]: 283 (100); [431 → 311 → 283, **MS** ^**4**^]: 241	Vitexin (apigenin‐8‐C‐glucoside)
7	31.20	287	[287, **MS** ^**2**^]: 269, 151 (100)	Aromadendrin
8	34.70	271	[271, **MS** ^**2**^]: 176, 151 (100)	Naringenin
9	38.90	269	[269, **MS** ^**2**^]: 267	Apigenin
10	39.20	299	[299, **MS** ^**2**^]: 283 (100); [299 → 283, **MS** ^**3**^]: 281 (100); [299 → 283 → 281, **MS** ^**4**^]: 267, 219 (100), 161	Trihydroxy‐methoxyflavone
11	41.40	313	[313, **MS** ^**2**^]: 298 (100); [313 → 298, **MS** ^**3**^]: 283 (100); [313 → 298 → 283, **MS** ^**4**^]: 255 (100), 239, 226, 183	Cirsimaritin
12	41.90	285	[285, **MS** ^**2**^]: 165 (100)	Sakuranetin
13	45.50	329	[329, **MS** ^**2**^]: 327, 285 (100); [329 → 285, **MS** ^**3**^]: 271 (100)	Hidroxytrimetoxiflavanone

### 3.2 Animal experiments and biochemical parameters

#### 3.2.1 Effect of ELo on body weight

Table 2 shows the effect of ELo on the body weight of alloxan‐induced hyperglycemic animals. The hyperglycemic animals decreased in body weight (−23.92%) within 2 weeks of induction compared to the normoglycemic animals. Oral treatment of ELo 75, 150, and 250 mg/kg for 15 days significantly increased body weight by 36.97%, 42.70%, and 43.94%, respectively, compared to hyperglycemic control animals.

**TABLE 2:** Effect of hydroalcoholic extract of *Lippia origanoides* Kunth (ELo) on body weight in alloxan‐induced hyperglycemic rats.

Days	NC	HC	ELo 75 mg/kg	ELo 150 mg/kg	ELo 250 mg/kg	Glib 5 mg/kg
0	226.62 ± 5.6	226.25 ± 3.7	228.63 ± 4.7	218.75 ± 4.5	220.50 ± 4.1	216,50 ± 3.0
7	242.25 ± 6.0^a^	204.00 ± 4.7	222.75 ± 6.1^a^	233.12 ± 4.4^a^	235.25 ± 4.2^a^	226,00 ± 3.3^a^
15	253.88 ± 7.3^a^	172.12 ± 4.5*	235.75 ± 7.5^*a^	245.62 ± 3.6^*a^	247,75 ± 4.8^*a^	234,50 ± 3.7^*a^

*Note*: All values are mean ± Std. Error (*n* = 10). Values with different superscripts are significantly different from each other at *p* < .05. A two‐way ANOVA changes according to Tukey's Multiple Range tests. Concerning *for NC (Normal Control) and ^a^for HC (Hyperglycemic Control).

Abbreviation: Glib, Glibenclamide group.

#### 3.2.2 Effects of ELo on the oral glucose tolerance test

The tolerance test was performed on hyperglycemic rats' control, treatment with ELo (150 mg/kg), and glibenclamide (5 mg/kg). The rats in the hyperglycemic control group had a significant elevation in blood glucose levels throughout the total measurement period (120 min). The treatment with ELo 150 mg/kg reduced the glucose level by 29.30% in comparison to the initial value. Of note, it did not come back to the initial value (0 min) even at the end of the period tested. The variation in blood glucose level of the glibenclamide group (5 mg/kg) was 17% reduced. Both treatments prevented the expressive increase in glucose levels observed in the hyperglycemic group after significant glucose overload (Table 3).

**TABLE 3:** Values of glucose in a tolerance test performed in alloxan‐induced hyperglycemic rats pretreated with a hydroalcoholic extract of *Lippia origanoides* Kunth (ELo).

Glucose time	HC	VG%	ELo 150 mg/kg	VG%	Glib 5 mg/kg	VG%
0 (min)	248.60 ± 8.02		315. 63 ± 12.22		347.13 ± 5.66	
30 (min)	378.00 ± 6.03	52.05	433.87 ± 8.22	37.46	448.22 ± 11.82	29.00
60 (min)	394.00 ± 7.22	58.48	344.00 ± 8.09	8.98	426.00 ± 8.14	22.72
90 (min)	406.40 ± 8.26	63.47	300.06 ± 6.79	4.93 (decrease)	319.00 ± 3.14	8.1 (decrease)
120 (min)	402.80 ± 10.33	62.03	223.12 ± 5.22	29.30 (decrease)	288.00 ± 3.0	17.03 (decrease)

*Note*: All values are mean ± Std. Error (*n* = 8).

Abbreviations: Glib, Glibenclamide group, HC, Hyperglycemic Control; NC, Normal Control; VG, Variation in blood glucose level.

#### 3.2.3 Effects of ELo treatment on fasting blood glucose in experimental groups

**FIGURE 3:**
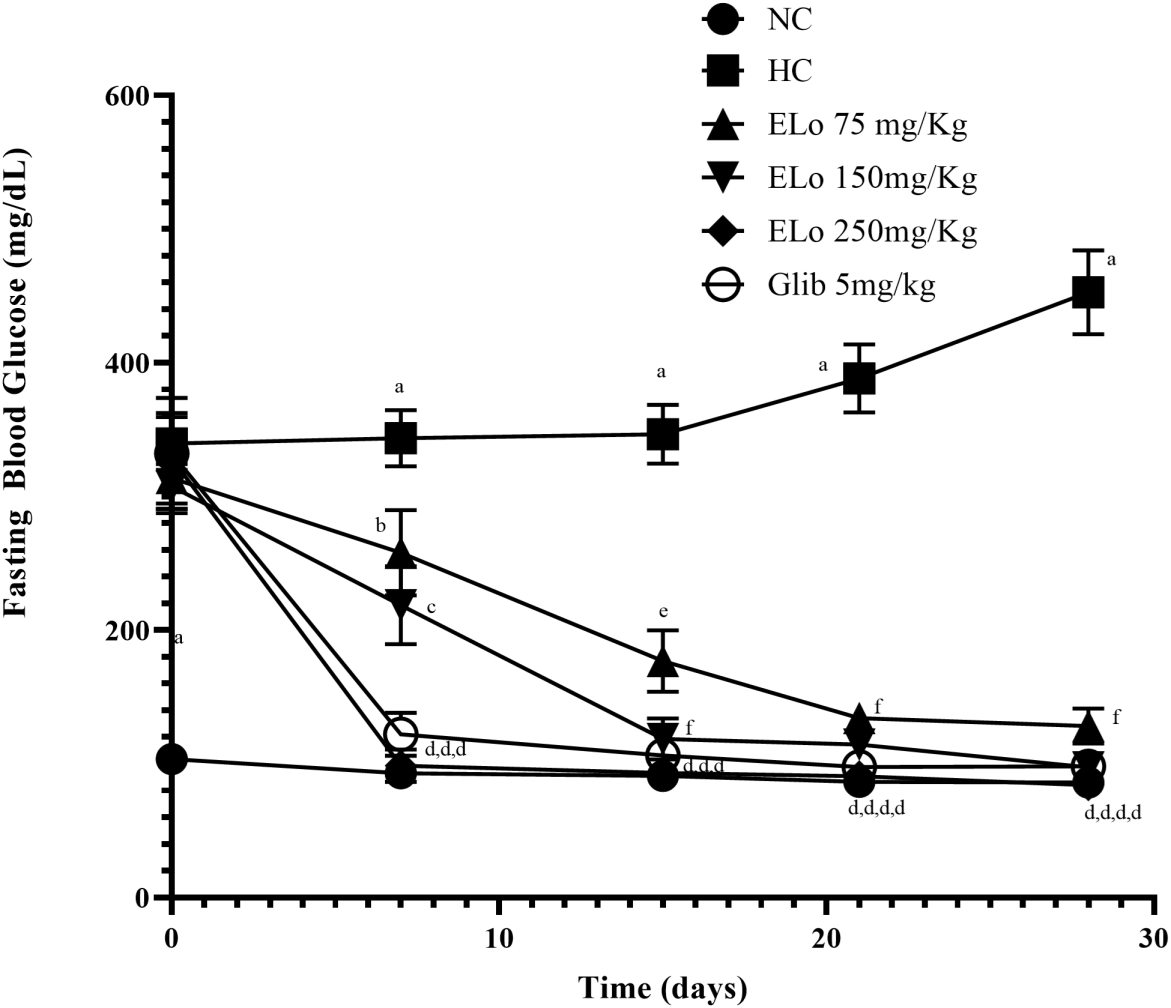
Effects of hydroalcoholic extract of *Lippia origanoides* Kunth (ELo) treatment on fasting blood glucose of the alloxan‐induced hyperglycemic rats. ELo treatment for 15 days (*n* = 10); 21 and 28 days (*n* = 5). Data are expressed as mean ± SEM. Values with different superscripts (a–h) are significantly different from each other at *p* < .001 and values with the same alphabet indications have insignificant changes according to Two‐way ANOVA and Tukey's Multiple Range tests.

Figure 3 illustrates the effects of ELo on the fasting blood glucose levels of the experimental groups. On day 3 post‐alloxan induction, there was a significant increase in fasting blood glucose in the entire group of alloxan‐induced hyperglycemic rats when compared to the normoglycemic group. Hyperglycemic rats treated with ELo (75, 150, and 250 mg/kg) and glibenclamide showed a decline in fasting blood glucose level from the 15th day of the treatment as compared to the hyperglycemic control group (Figure 3). Treatment of ELo 250 mg/kg (93.13 ± 2.69 mg/dL) in the hyperglycemic rats significantly brought the blood glucose values to the normal level as compared to the normal control group (89.17 ± 2.7 mg/dL) on the 15th day of the treatment. Figure 3b shows that the effect is present for other doses (75 mg/kg and 150 mg/kg) and even greater when treatment with 250 mg/kg is maintained for 28 days.

#### 3.2.4 Effect of ELo lipid profile

Serum levels of total cholesterol, triacylglycerides, and LDL cholesterol were significantly higher in hyperglycemic control rats than in the normoglycemic group, while HDL cholesterol levels were considerably reduced. Continuous treatment with ELo for 15 days produced a significant improvement in the deteriorating effects of the hyperglycemic state on cholesterol, triacylglycerides, LDL, and HDL values. ELo 250 mg/kg was the most effective in attenuating cholesterol, triacylglycerides, and LDL (mg/dl) levels in hyperglycemic rats, as well as increasing HDL levels (Table 4). The atherogenic index and the atherogenic coefficient were significantly elevated in hyperglycemic control when compared to normal control and were reduced by all different ELo treatments (Table 4). Compared to the normoglycemic group (3.57 ± 0.33), the cardiovascular risk index was higher in the hyperglycemic control (6.93 ± 0.24). ELo reduced these indices at all studied doses (Table 4). The reduction presented by the extract treatments was greater than that presented by the glibenclamide group.

**TABLE 4:** Effect of hydroalcoholic extract of *Lippia origanoides* Kunth (ELo) on the lipid profile, atherogenic risk, and cardiovascular risk in alloxan‐induced hyperglycemic rats.

	NC	HC	Elo 75 mg/kg	Elo 150 mg/kg	Elo 250 mg/kg	Glib 5 mg/kg
Triacyglycerides (mg/dl)	100.40 ± 3.20	159.2 ± 2.90^#^	126.00 ± 5.8^*#^	120.80 ± 2.20*	115.20 ± 6.70*	148.40 ± 7.9^#^
Total cholesterol (mg/dl)	52.80 ± 3.80*	65.40 ± 3.30^#^	59.00 ± 4.20^#^	51.80 ± 2.10*	51.40 ± 2.60*	59.80 ± 2.20^#^
HDL (mg/dl)	14.90 ± 0.70	9.458 ± 0.50^#^	11.25 ± 0.30^#^	12.76 ± 0.30^#*^	13.91 ± 0.50*	12.14 ± 0.40^#*^
LDL (mg/dl)	17.80 ± 3.40	24.10 ± 2.7^#^	22.55 ± 4.70	14.88 ± 2.10*	14.45 ± 2.90*	17.98 ± 3.20*
Atherogenic Index	2.49 ± 0.36*	5.92 ± 0.23^#^	4.28 ± 0.48^#^	3.06 ± 0.15*	2.92 ± 0.33*	3.93 ± 0.18^#*^
Atherogenic Coefficient	1.21 ± 0.26	2.53 ± 0.24^#^	2.03 ± 0.46	1.16 ± 0.16*	1.04 ± 0.22*	1.46 ± 0.25*
Cardiovascular Risk Index	3.57 ± 0.33*	6.93 ± 0.24^#^	5.28 ± 0.48^#*^	4.06 ± 0.14*	3.71 ± 0.21*	4.94 ± 0.19^#*^

*Note*: All values are mean ± Std. Error (*n* = 5). *Concerning HC (Hyperglycemic Control), ^#^to NC (Normal Control) and Glib (Glibenclamide group). Values with different superscripts are significantly different from each other at *p* < .05. A two‐way ANOVA changes according to Tukey's Multiple Range tests.

#### 3.2.5 Effect of ELo liver, renal function parameters, and plasma MDA level

As displayed in Table 5, the serum AST, ALT, and plasma MDA levels of hyperglycemic control rats were significantly higher when compared to the normoglycemic group. Of all the doses, ELo at 250 mg/kg revealed the maximum reduction of AST, ALT, and MDA levels in the hyperglycemic and glibenclamide groups. As shown in Table 5, the hyperglycemic rats showed a pronounced increase in their urea and creatinine levels compared to the normoglycemic group. The kidney parameters of serum urea and creatinine have been reduced for ELo treatment.

**TABLE 5:** Effect of hydroalcoholic extract of *Lippia origanoides* Kunth (ELo) on liver and kidney function and oxidative stress in alloxan‐induced hyperglycemic rats.

	NC	HC	ELo 75 mg/kg	ELo 150 mg/kg	ELo 250 mg/kg	Glib 5 mg/*
AST (U/L)	113.7 ± 3.3	218.7 ± 5.2^#^	127.4 ± 9.6*	109.8 ± 4.9*	107.6 ± 5.9*	116.0 ± 4.8*
ALT (U/L)	27.1 ± 1.6	35.9 ± 1.7^#^	31.9 ± 0.9	27.5 ± 1.2*	26.34 ± 1.4*	34.83 ± 0.8^#^
Creatinine (mg/dl)	0.58 ± 0.03	0.78 ± 0.03^#^	0.70 ± 0.03	0.63 ± 0.03*	0.56 ± 0.03*	0.71 ± 0.03^#^
Urea (mg/dl)	65.29 ± 1.08	204.4 ± 4.5^#^	164 ± 8.14^#*^	148.6 ± 4.03^#*^	153.9 ± 6.72^#*^	159.0 ± 5.7^#*^
MDA (μM)	54.5 ± 1.4	444.4 ± 22.3^#^	224.0 ± 13.8^#*^	141.5 ± 7.4^#*^	69.0 ± 5.6*	236.7 ± 15.96^#*^

*Note*: All values are mean ± Std. Error (*n* = 5). *Concerning HC (Hyperglycemic Control), ^#^to NC (Normal Control) and Glib (Glibenclamide group). Values with different superscripts are significantly different from each other at *p* < .05. A two‐way ANOVA changes according to Tukey's Multiple Range tests.

#### 3.2.6 Effect of ELo on insulin and HbA1c levels

**FIGURE 4:**
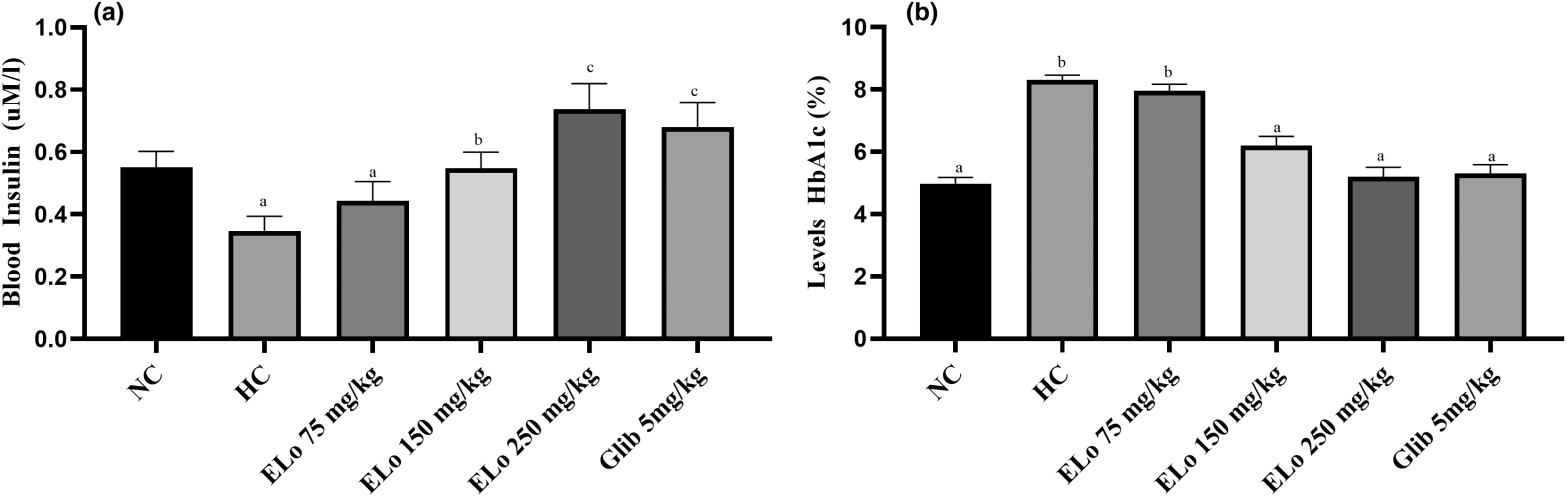
(a,b) Effects of hydroalcoholic extract of *Lippia origanoides* Kunth (ELo) treatment on insulin and glycated hemoglobin (HbA1c) levels of the alloxan‐induced hyperglycemic rats. ELo treatment for 28 days (*n* = 5). Data are expressed as mean ± SEM. Values with different superscripts (a–c) are significantly different from each other at *p* < .05 and values with the same alphabet indications have insignificant changes according to Two‐way ANOVA and Tukey's Multiple Range tests.

Figure 4a,b illustrates the effects of ELo on insulin levels, HbA1c, and fasting blood glucose levels. Treatment of hyperglycemic rats with ELo and glibenclamide significantly increased the insulin level after 28 days when compared to hyperglycemic control rats in a dose‐dependent manner (0.347 ± 0.021); ELo 75, 150, and 250 mg/kg show 0.444 (±0.027), 0.548 (±0.023)*, and 0.738 (±0.036)*# μM/l, respectively. From ELo treatment with 150 mg/kg, the insulin values did not differ statistically from normoglycemic rats (0.550 ± 0.023) uM/L. ELo 250 mg/kg promoted greater insulin release than the glibenclamide group (0.680 ± 0.035) (Figure 4a).

The levels of HbA1c (%) were higher in hyperglycemic rats (8.30 ± 0.161)* than in normoglycemic rats (4.98 ± 0.196) (*p* < .05). However, there was a reduction in HbA1c levels with treatment with ELo at doses of 150 mg/kg (6.20 ± 0.295)* and ELo 250 mg/kg (5,2 ± 0.300)*, not revealing statistically different from the group Glib 5 mg/kg (5.3 ± 0.286)* (Figure 4b).

#### 3.2.7 Relative activity of α‐glucosidase in the presence of ELo

**FIGURE 5:**
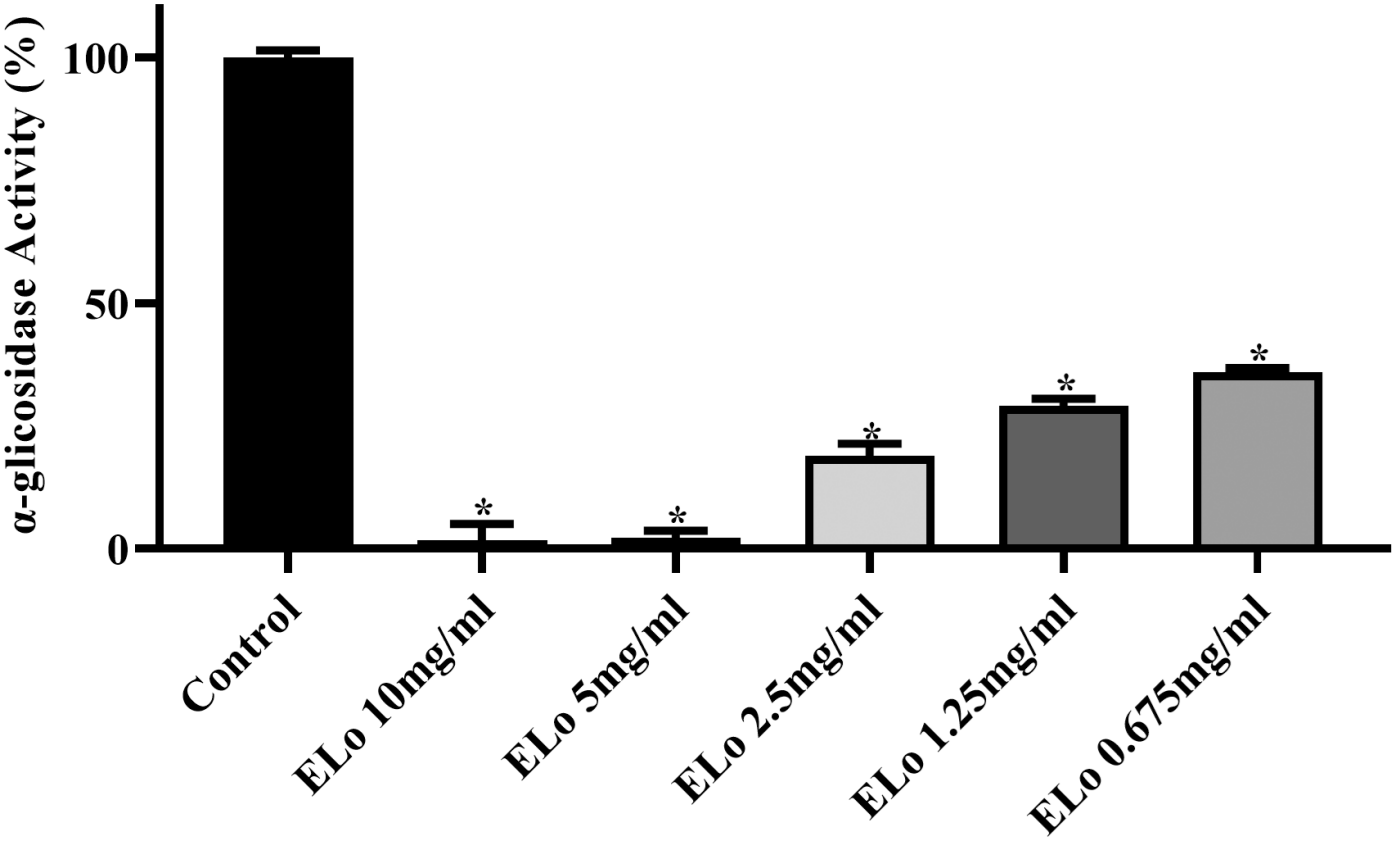
Effect of the hydroalcoholic extract of *Lippia origanoides* Kunth (ELo) on the relative activity of α‐glucosidase in vitro. The ELo dilutions (10.0–0.675 mg/mL) show the inhibitory activity of α‐glucosidase. Two‐way ANOVA or Dunnett's post hoc test *p < .05.

The ELo dilutions (10.0–0.675 mg/mL) showed the inhibitory activity of α‐glucosidase in vitro (Figure 5). The anti‐α‐glucosidase activity was in the range of 98.61%–64.09%.

### 3.3 Histopathological findings

**FIGURE 6:**
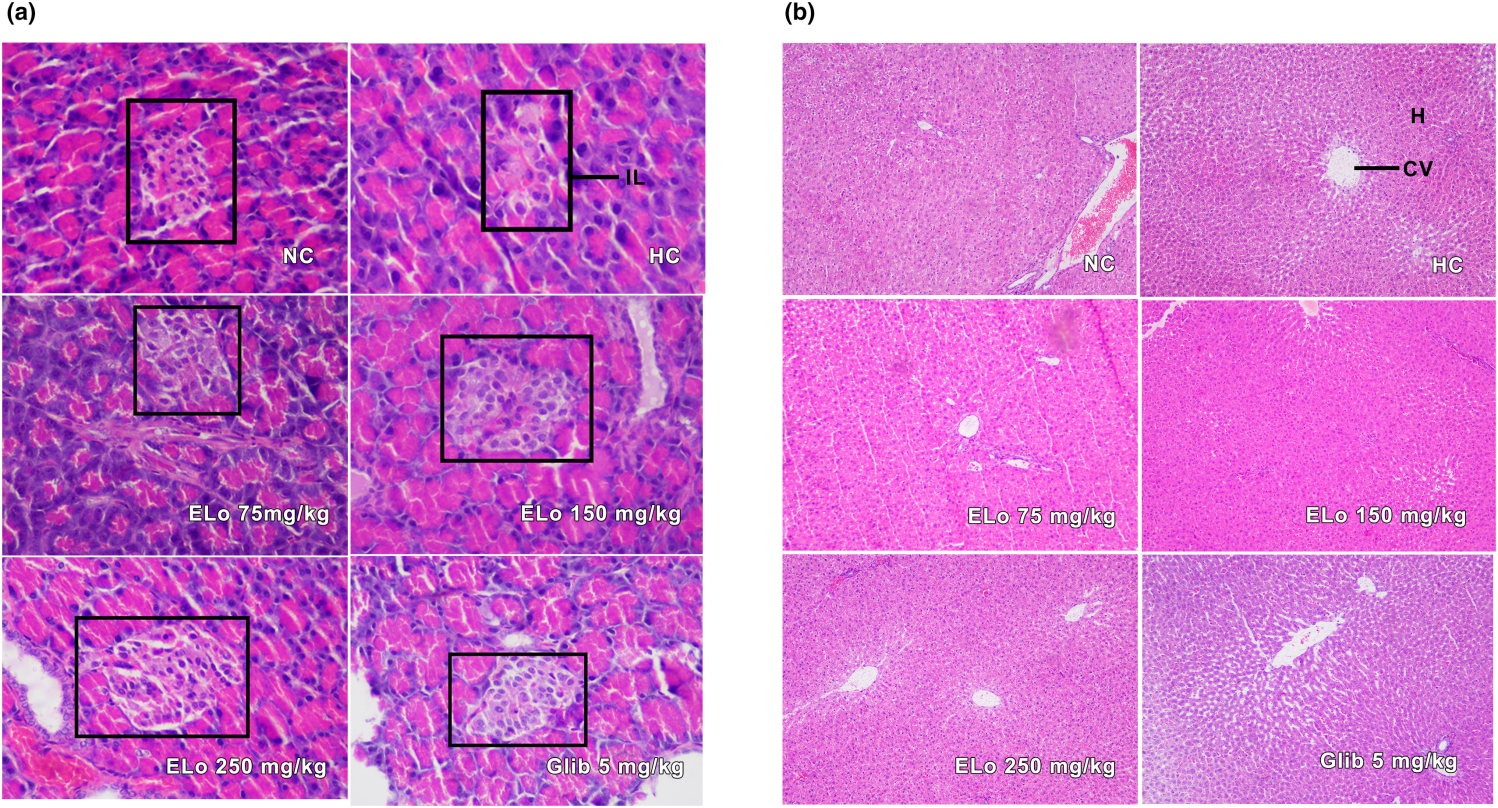
(a,b) Histopathological observations of the pancreas (a) and liver tissues (b) from alloxan‐induced hyperglycemic rats after treatment with a hydroalcoholic extract of *Lippia origanoides* Kunth (ELo). Photomicrographs (40X). (a) presents pancreatic β‐cells and the islet tissue section (IL). (b) shows the liver tissue sections (CV, Central vein; H, hepatocytes). For both images: ELo 75, 150 at 250 mg/kg; Glib, Glibenclamide group; HC, Hyperglycemic Control; NC, Normal Control.

Histological observation of the islet cells of the normal control group showed normal acini and islet cells with no structural changes. Histology of the islet cells of the hyperglycemic control showed degeneration and extensive necrotic changes in the islet cells, followed by atrophy, in comparison with the normal control. Treatment with ELo showed visible positive changes in the histoarchitecture of pancreatic β‐cells. The islet tissue section of the glibenclamide‐treated group also showed a better appearance of β‐cells, as evidenced by the histopathological observation (Figure 6a). The liver section of the alloxan‐induced untreated hyperglycemic group showed congestion of the central veins and hepatocellular necrosis with vacuolization compared to that of the normoglycemic group. However, hyperglycemic rats treated with ELo and glibenclamide effectively reduced the severity of degenerative changes by attenuating the hepatic necrosis and reversing the hepatic architecture. Histology of the normoglycemic group showed normal hepatocytes and central veins (Figure 6a,b).

## 4 DISCUSSION

In some medicinal plants, phenolic compounds are responsible for reducing blood glucose levels (Kumar et al., 2021). Therefore, the hypoglycemic effects demonstrated by ELo may be associated with the majority of flavonoids in the chemical composition of the species (Table 1). Oliveira et al. (2014) also described the link between glucose uptake and metabolism stimulation, promoted by the presence of these compounds in the species *L. origanoides* Kunth.

In this context, within the *Lippia* genus, *L. nadiflora* (Balamurugan & Ignacimuthu, 2011) and *L. javanica* (Arika et al., 2015) are described with promising evidence of hypoglycemic action, being plant species that present phenolic compounds in common with several flavonoids identified in the present study. This suggests the synergistic action of these substances as a possible trigger for the antihyperglycemic effect of ELo, as of the thirteen flavonoids identified in the extract (Table 2), nine had already demonstrated well‐founded hypoglycemic effects in previous studies: Naringenin, Apigenin, Vicenin‐2, Cirsimaritin, Orientin, Sakuranetin, Aromadendrin, Vitexin, and Isorhamnetin‐3‐O‐rutinoside.

Naringenin and its glycosides found in citrus fruits, for example, have antidiabetic activity (Boudjelal et al., 2015; Nyane et al., 2017). The presence of Naringenin and Cirsimaritin in *Lippia graveolens* was associated with the inhibitory activities of the enzymes DPP‐IV (which degrades GLP‐1, glucagon‐like peptide 1) and PTP1B (protein tyrosine phosphatase 1B, a negative regulator of insulin signal transduction), having a hypoglycemic effect (Bower et al., 2014). GLP‐1 is a potent anti‐hyperglycemic that induces stimulation of glucose‐dependent insulin secretion while suppressing glucagon secretion (the hormone opposite to insulin) (Ramracheya et al., 2018). DPP‐IV inhibitors are already used mainly as adjuvants in the treatment of diabetes, and the search for PTP1B modulators, such as Vicenin‐2, has attracted increasing attention as a therapeutic target in the treatment of diabetes and obesity (Islam et al., 2014; Liu, Mathieu, et al., 2022).

DPP‐IV enzyme inhibitors have successfully reduced hyperglycemia and HbA1c levels in monotherapy and in combination with other antidiabetic agents (Wu et al., 2014). HbAC1 is a biochemical marker associated with monitoring diabetes; its elevation is related to chronic hyperglycemic states (Stolar, 2010). The values of HbAC1 were reduced in the groups treated with ELo (150 and 250 mg/kg) and with Glibeclamide (5 mg/kg).

Preliminary clinical trials have suggested that GLP‐1 agonists may also reduce the risk of liver damage associated with diabetes (Wester et al., 2024). ELo treatment also showed a reduction in serum ALT and AST levels compared to the hyperglycemic control group. The significant increase in serum ALT and AST levels in the untreated hyperglycemic animals showed the extent of liver damage, which demonstrates the impairment of liver function related to hyperglycemia. Therefore, the reduction of these biomarkers to levels closer to normal indicates a decrease in hyperglycemic complications in the groups treated with ELo.

It is also worth highlighting the agreement of these results with the histological findings. The liver histopathological examinations in the hyperglycemic control group showed damaged hepatocytes. Treatment with ELo revealed a remarkable improvement in the histoarchitectural alterations, indicating the healing potential. This finding is consistent with previous literature (Rodríguez et al., 2018), where Naringin demonstrated a hepatoprotective role in the diabetes model, preventing histological changes in the liver of treated animals by blocking oxidative/nitrosative stress.

GLP‐1 receptor analogs have also shown promise in slowing the progression of diabetic nephropathy (Bonner et al., 2020). Studies describe deleterious effects on kidney function in the context of diabetes (Alicic et al., 2017; Kene et al., 2021; Liu, Ma, et al., 2022), with diabetes being one of the main causes of kidney failure (Kene et al., 2021). Diabetic nephropathy is represented by an abnormality in markers of renal function (urea and serum creatinine), and treatment with ELo revealed a reduction in the serum values of these markers, concerning those expressed by the hyperglycemic control group, and suggested a reduction in associated renal failure with complications of diabetes due to the inhibition of protein degradation.

Orientin is another component of ELo with the potential to be used in the treatment of diabetic nephropathy. Other findings (Kong et al., 2020) indicate that Orientin significantly reduces the rate of early apoptosis of podocyte cells (renal epithelium). In the face of autophagy disorders induced by high hyperglycemia, this flavone protects podocytes by protecting the integrity of the mitochondrial membrane. It was also by inhibiting a cell death mechanism that Vitexin demonstrated potential in the treatment of rats with diabetic nephropathy, by attenuating renal fibrosis, oxidative damage, and ferroptosis through the activation of GPX4 (glutathione peroxidase 4) (Zhang et al., 2023), revealing yet another indicator of Elo's multi‐action pathway.

Returning to possible mechanisms associated with hypoglycemic effects, several compounds present in Elo have been shown to improve insulin resistance by regulating the glucose transporters GLUT2 and GLUT4. A reduction in these glucose transporters is observed in diabetes (Malik et al., 2019). Naringenin increases the expression of insulin receptor messenger RNA and GLUT4 β subunits, increasing the production of these transporters and receptors, causing insulinotropic effects (increased insulin secretion and consequent decrease in glucose levels) (Ahmed et al., 2017; Den Hartogh & Tsiani, 2019). In diabetic mice, free isorhamnetin performed as well as standard treatment in regulating GLUT4 expression and increasing p‐AMPK‐α expression (Alqudah, Qnais, et al., 2023). Similarly, Cirsimaritin, in addition to offering antioxidant and anti‐inflammatory effects, positively regulated the activation of the GLUT4‐AMPK and GLUT2‐AMPK pathways (responsible for glucose uptake and necessary for the physiological control of glucose‐sensitive genes) in skeletal muscle, adipose tissue, and the liver (Alqudah, Athamneh, et al., 2023).

Activation of AMP‐activated protein kinase (AMPK) increases glucose uptake through an insulin‐independent pathway by stimulating the translocation of GLUT4 and GLUT2 to the cell surface (Dayarathne et al., 2021; Giacoman‐Martínez et al., 2021; Vlavcheski et al., 2020). When phosphorylated (p‐AMPK‐α), this protein improves the use of glucose (Rena et al., 2013), contributing to the reduction of hyperglycemia. In in vitro models, Orientin demonstrated the ability to increase glucose uptake and effectively modulate the expression of genes that encode glucose transport (GLUT2 and GLUT4) (Choi et al., 2020; Mazibuko‐Mbeje et al., 2021).

The hyperglycemic state deteriorates the body's antioxidant defense mechanism, which consequently increases MDA levels and reduces the activity of antioxidant enzymes (Wang & Wang, 2017). The loss of β‐cells through apoptosis reduces insulin production, which contributes to the onset and progression of diabetes. The rate of generation of oxygen species (ROS) can act directly on glycemic control since the antioxidant potential of natural products promotes a decrease in the generation of ROS and, thus, protects β‐cells and improves their function (Mojica et al., 2017).

The protection of the pancreatic β‐cells may keep insulin release closer to that of normoglycemic individuals. Apigenin attenuates pancreatic β‐cell injury in animals with chemically induced diabetes, not only as a free radical scavenger but also by enhancing the antioxidant enzymatic system of these cells (Wang et al., 2017). This antioxidant capacity is usually associated with phenolic compounds. Vitexin defends pancreatic β‐cells from damage induced by high glucose toxicity, possibly through the increase in intracellular antioxidant molecules and suppression of the inflammatory signaling pathway, as consequences of increased expression of the Nrf2 transcription factor (nuclear factor erythroid 2‐related factor 2) (Ganesan et al., 2020). Therefore, ELo's ability to reduce MDA levels demonstrates its potential in the face of hyperglycemia‐related oxidative stress.

In addition to significantly reducing MDA levels, Naringenin and Apigenin reduce ICAM‐1 (intracellular adhesion molecule 1), which may directly improve the vascular reactivity response (Ren et al., 2016). In this regard, in vitro and in vivo, Vicenin‐2 was able to suppress vascular inflammatory processes induced by high glucose levels (Ku & Bae, 2016).

Reducing ICAM‐1 may have a direct effect on improving the hardening or narrowing of arteries, as its overexpression is associated with increased ROS generation, which promotes endothelial dysfunction, the initial stage of atherosclerosis (Galkina & Ley, 2007; Habas & Shang, 2018). Therefore, since ICAM‐1 is related to the development of cardiovascular diseases, Apigenin, Naringenin, and Vicenin‐2 can be considered cardioprotective in heart diseases involving diabetic animals (Ku & Bae, 2016; Liu et al., 2017; Mahajan et al., 2017; Ren et al., 2016). This corroborates the reducing action of ELo on the predictive indices of atherogenic risk, also reducing the cardiovascular risk factor “dyslipidemia.” In this context, using plant antioxidants can lead to protective effects against the formation and progression of atherogenicity (Rafieian‐Kopaei et al., 2013).

Another possible mechanism of action of Elo in cardiac dysfunction associated with diabetes is through the modulation of genes that encode insulin signaling. Elo compounds (Orientin and Vitexin) capable of activating insulin receptor substrate‐1 (IRS‐1), in addition to increasing glucose uptake, play an important role in the activation of phosphatidylinositide‐dependent kinase‐3 (PI‐3K) by increasing the phosphorylation of IR, IRS‐1, and IRS‐2 (Choi et al., 2020; Ganesan et al., 2020; Inamdar et al., 2019; Mazibuko‐Mbeje et al., 2021). PI‐3K controls the signaling cascade that regulates mitochondrial function, cardiac energy metabolism, and the renin–angiotensin system (Guo & Guo, 2017). Therefore, controlling metabolic and cardiovascular diseases can benefit from therapeutic strategies that regulate these signaling cascades affected by insulin resistance.

Lipid abnormalities that contribute to the onset of cardiovascular diseases, including increased levels of total cholesterol, triacylglycerides, LDL, and decreased levels of HDL, are commonly associated with diabetes. Diabetic patients are more likely to have cardiovascular diseases due to dyslipidemia (Einarson et al., 2018; Wu & Parhofer, 2014). The alteration of the lipid profile in diabetes is linked to the elevated hepatic production of triglyceride‐rich lipoproteins, leading to increased formation of atherogenic very low‐density lipoproteins (Ivanova et al., 2017). However, in the present study, lipid profiles were decreased in the ELo‐treated groups and upregulated in the hyperglycemic control group. A positive correlation was observed between hyperglycemia and dyslipidemia. Continuous treatment at three different doses of ELo revealed significant hypoglycemic and lipid‐lowering effects. This may be due to the possible stimulatory effect of the extracts on the remaining β‐cells, increasing secretion, responsiveness, or insulin‐mimicking activity. In agreement with our findings, free isorhamnetin had a positive effect on the dyslipidemia present in diabetic mice, significantly reducing the levels of serum triglycerides, total cholesterol, and LDL compared to the control group (Alqudah, Qnais, et al., 2023).

Several plant extracts with anti‐hyperglycemic potential present enzymatic regulation of carbohydrate metabolism, glucose homeostasis, and protection of pancreatic β‐cells (Ullah et al., 2020). Sakuranetin and Aromadendrin can increase adipogenesis and insulin sensitivity of 3 T3‐L1 adipocytes by regulating the peroxisome‐activated receptor γ2 (PPARγ2) (Saito et al., 2008; Zhang et al., 2011), stimulating glucose absorption in these cells, and thus sensitizing insulin adipocytes. This regulation may also be related to the lower weight loss shown by hyperglycemic animals treated with Elo compared to hyperglycemic animals without treatment, improving general clinical conditions.

The Alloxan model of hyperglycemia could result in the partial (or total, dose‐ and time‐dependent) destruction of the β‐cells of the pancreas and a decline in serum levels of insulin because of decreased synthesis. The actions of the ELo in reducing hyperglycemia (Figure 3) and maintaining insulin levels close to those of normoglycemic animals and the glibenclamide group within 28 days suggest activation of β‐cells in the pancreas of hyperglycemic rats (Figure 4a). A similar profile is presented by the histological findings (Figure 6a), where an attenuation in the reduction of the area of the pancreatic islets is observed in the animals submitted to treatment with ELo concerning the hyperglycemic control group (Figure 6b). A methanolic extract obtained from a plant of the same genus as ELo (*L. nadiflora*) acted similarly to reduce the cellular atrophy of pancreatic islets in diabetic rats compared to the untreated control group (Balamurugan & Ignacimuthu, 2011).

At the intestinal level, the α‐glucosidase enzyme directly participates in the breakdown of carbohydrates such as sucrose, starch, and maltose, favoring digestion and increasing postprandial glycemia (Baron, 1998). An essential inhibitory rate of α‐glucosidase was obtained by optimized methanolic and supercritical extracts of *L. graveolens* leaves (Picos‐Salas et al., 2021), identifying many flavonoids whose inhibitory activity is related to their hydroxylation and gallolylation in these compounds, as well as the presence of an unsaturated 2,3 bond in conjugation with a 4‐carbonyl group (Xiao et al., 2013). In this context, compounds such as Vicenin‐2 and Vitexin and derivatives (isovitexin‐4′‐methyl ether, isovitexin, and 2″‐o‐xylopyranosyl vitexin) can act as natural hypoglycemic agents due to their potent α‐glucosidase inhibitory activity (Chen et al., 2013; Choo et al., 2012; Islam et al., 2014; Li et al., 2009; Shibano et al., 2008; Yang et al., 2016; Yao et al., 2011). Likewise, isorhamnetin‐3‐O‐rutinoside showed good binding with α‐glucosidase when subjected to molecular docking (Sabiu et al., 2021). Therefore, in addition to the effects of ELo on glucose metabolism and antioxidant activity, the results suggest that the hypoglycemic action of the extract may be related to its ability to inhibit this enzyme (Figure 5).

The protective action of ELo on the different metabolic parameters evaluated reveals the multi‐active profile of this extract in the context of hyperglycemia. The compilation of the studies discussed strengthens the presence of a synergistic action of the substances present in Elo, indicating that the doses analyzed have potential anti‐hyperglycemic properties, a lipid‐lowering cardiovascular protective effect, and attenuating effects on liver and kidney parameters, factors that directly contribute to a general improvement in diabetes.

## 5 CONCLUSION

The present study revealed that *L. origanoides* Kunth has shown hypoglycemic effects and protective effects on liver, kidney, and lipid profiles. Phytochemistry analysis of ELo indicated that it contains a considerable number of flavonoids that affect controlling blood glucose levels, such as Naringenin, Apigenin, Vicenin‐2, Cirsimaritin, Orientin, Sakuranetin, Aromadendrin, Vitexin, and Isorhamnetin‐3‐O‐rutinoside. In all doses, this extract significantly reduced the hyperglycemic and total cholesterol, triacylglycerides, LDL and atherogenic index, atherogenic coefficient, and cardiovascular risk index in diabetic rats. The ELo dose of 250 mg/kg showed the maximum reduction in AST, ALT, and MDA when compared to the hyperglycemic and glibenclamide groups. Possible mechanisms of action may be the stimulation of insulin release from remaining pancreatic β‐cells, the inhibitory activity of α‐glucosidase, improving general clinical conditions by minimizing the weight loss characteristic of diabetes induction, and the antioxidant effects of the extract.

The proposal's limitation is that it does not determine the exact contribution of each compound to the pharmacological actions presented. However, this study aims to analyze the action of an extract and not the action of isolated compounds, since the synergism between these agents can extend their therapeutic applicability. This promising activity found in ELo encourages the application of future toxicological tests to investigate possible toxic effects of the extract, opening perspectives for the development of a phytotherapeutic presentation of *L. origanoides* Kunth as a hypoglycemic and cardiovascular protective lipid‐lowering effect.

## AUTHOR CONTRIBUTIONS

**Vinicius Carvalho Miranda:** Conceptualization (equal); investigation (equal); writing – original draft (supporting). **Yago Luis Gonçalves Pereira:** Investigation (equal); methodology (equal); writing – original draft (equal); writing – review and editing (supporting). **Allane Patricia Santos da Paz:** Investigation (equal); methodology (equal); writing – original draft (supporting); writing – review and editing (equal). **Keyla Rodrigues de Souza:** Investigation (equal); methodology (equal). **Márcia Cristina Freitas da Silva:** Investigation (equal); methodology (equal). **Nilton Akio Muto:** Investigation (equal); methodology (equal). **Patrick Romano Monteiro:** Investigation (equal); methodology (equal). **Agenor Valadares Santos:** Investigation (equal); methodology (equal). **Moises Hamoy:** Data curation (equal); formal analysis (equal). **Maria das Graças Freire de Medeiros:** Data curation (equal); formal analysis (equal). **Iolanda Souza do Carmo:** Investigation (equal); methodology (equal). **Maria Eduarda Moraes Silva:** Investigation (equal); methodology (equal). **José de Sousa Lima Neto:** Conceptualization (equal); investigation (equal); methodology (equal). **Vanessa Jóia de Mello:** Conceptualization (lead); investigation (lead); methodology (lead); writing – original draft (equal); writing – review and editing (lead).

## FUNDING INFORMATION

The APC was funded by the Pró‐Reitoria de Pesquisa e Pós‐graduação da Universidade Federal do Pará (PROPESP/UFPA/PAPQ).

## CONFLICT OF INTEREST STATEMENT

The authors declare that the research was conducted in the absence of any commercial or financial relationships that could be construed as a potential conflict of interest.

## Data Availability

All corresponding data are presented in this study.
